# Reuse of Ti6Al4V Powder and Its Impact on Surface Tension, Melt Pool Behavior and Mechanical Properties of Additively Manufactured Components

**DOI:** 10.3390/ma14051251

**Published:** 2021-03-06

**Authors:** Mateusz Skalon, Benjamin Meier, Thomas Leitner, Siegfried Arneitz, Sergio T. Amancio-Filho, Christof Sommitsch

**Affiliations:** 1 IMAT Institute of Materials Science, Joining and Forming, Graz University of Technology, Kopernikusgasse 24, 8010 Graz, Austria; mateusz.skalon@tugraz.at (M.S.); sergio.amancio@tugraz.at (S.T.A.-F.); christof.sommitsch@tugraz.at (C.S.); 2 Joanneum Research, Materials—Institute for Laser and Plasma Technology, Leobner Straße 94, 8712 Niklasdorf, Austria; benjamin.meier@joanneum.at; 3 Institute of Experimental Physics, Graz University of Technology, NAWI Graz, Petersgasse 16, 8010 Graz, Austria; thomas.leitner@tugraz.at

**Keywords:** Ti6Al4V, LB-PBF, SLM, 3D printing, powder, reuse, melt pool, density, electromagnetic levitation, surface tension, reuse, powder

## Abstract

The quality and characteristics of a powder in powder bed fusion processes play a vital role in the quality of additively manufactured components. Its characteristics may influence the process in various ways. This paper presents an investigation highlighting the influence of powder deterioration on the stability of a molten pool in a laser beam powder bed fusion (LB-PBF, selective laser melting) process and its consequences to the physical properties of the alloy, porosity of 3D-printed components and their mechanical properties. The intention in this was to understand powder reuse as a factor playing a role in the formation of porosity in 3D-printed components. Ti6Al4V (15 μm–45 μm) was used as a base material in the form of a fresh powder and a degraded one (reused 12 times). Alloy degradation is described by possible changes in the shape of particles, particle size distribution, chemical composition, surface tension, density and viscosity of the melt. An approach of 3D printing singular lines was applied in order to study the behavior of a molten pool at varying powder bed depths. Single-track cross-sections (STCSs) were described with shape parameters and compared. Furthermore, the influence of the molten pool stability on the final density and mechanical properties of a material was discussed. Electromagnetic levitation (EML) was used to measure surface tension and the density of the melt using pieces of printed samples. It was found that the powder degradation influences the mechanical properties of a printed material by destabilizing the pool of molten metal during printing operation by facilitating the axial flow on the melt along the melt track axis. Additionally, the observed axial flow was found to facilitate a localized lack of fusion between concurrent layers. It was also found that the surface tension and density of the melt are only impacted marginally or not at all by increased oxygen content, yet a difference in the temperature dependence of the surface tension was observed.

## 1. Introduction

Progress in powder-based additive manufacturing (AM) such as laser beam powder bed fusion (LB-PBF) has reached process maturity in the past few years. These processes are being continuously developed further due to their multiple advantages and unique characteristics [1]. LB-PBF of a material of nearly full relative density currently no longer poses a technological barrier for most of alloys [2,3,4]. Unfortunately, this new production technique has to deal with some frequently observed technical problems. The relative density of a printed part often varies between 99.1% and 99.9% [2,3]. For this reason, previous investigations have focused mainly on the influence of process parameters on the relative density and mechanical properties [2,3,5]. None of them, however, managed to reach relative densities higher than 99.9%. This implies a residual porosity being always present, lowering the mechanical performance of an LB-PBF-printed part, especially in tensile mode or in fatigue-related applications [4,6,7]. Porosity creation in LB-PBF-fabricated samples is a complex process, but the main role in this, however, is currently assigned either to the quality of the powder material, the powder particles’ morphology, inadequate process parameters, instability of the melt pool or the influence of spatters [8,9,10,11,12,13,14,15,16,17]. Thijs et al. [3] stated that spherical pores were formed by powder denudation accumulation around the melt pool within a layer and an accumulation of surface roughness across the layers. Qiu, Adkins and Attallach [9] have observed open pores on the top surfaces of samples. They stated that near-spherical pores found in laser-processed Ti6Al4V alloys were due to incomplete re-melting of some localized surface areas of the previous layer and to the insufficient feeding of molten metal to solidification fronts. Das [10], on the other hand, suggested that the formation of flat or irregular-shaped pores originates from incomplete homologous melting and solidification. Additionally, Panwisawas et al. [18], using computational fluid dynamics simulation, proved that porosity may be created by the negative change in flow pattern in the melt pool, which is dictated by forces such as vapor pressure, gravitational force and capillary and thermal capillary forces exerted on the metallic/gaseous interface. This observation is in a good agreement with observations of Thijs et al. [3] who observed pores that were elongated along the scanning path. The state-of-the-art research did not take into consideration the technological characteristics of the powder and considered it as a source of a porosity. Moreover, none of the previous works focused on the physical simulation of a melt pool in order to study its behavior. Published investigations indicate that along the printing process, the feedstock powder loses its original characteristics, resulting in increased thickness of oxides layer on particles’ surfaces [19] and/or particles becoming more irregular [16]. Therefore, an in-depth investigation needs to performed in order to explain the origins of the porosity and to either minimize or prevent it by understanding the fundamental mechanisms that are its cause. The current research was aimed at understanding a possible link between the reuse of a Ti6Al4V powder and the characteristics and performance of the powder. The investigation was focused on intake of gaseous elements, surface tension, stability of a melt pool, porosity and mechanical properties.

The research approached the problem by modifying a well-established approach of printing singular tracks, which is commonly used for describing a molten pool, its behavior and material properties in specific processing conditions [20,21,22,23,24,25,26,27]. As indicated by Hu et al. [20], a standard approach may be incorrect (i.e., for wetting angle) due to the dynamic behavior of a molten pool that may easily become unstable. This method was modified in this work so that 90 lines were printed under given conditions, while each line was printed with an incrementally increasing thickness of the powder layer, to avoid the aforementioned limitation. This approach allowed the obtention of a population of single-track cross-sections (STCSs) showing how the singular track behaves under real-life conditions when the powder layer depth varies, mimicking the real LB-PBF process. Using the aforesaid population of STCSs, a thorough analysis of molten track behavior was performed in order to study and explain the influence of powder degradation on printing results. This, linked with a final density and a mechanical performance, allowed the description of the mechanism and indicated the origins of the appearance of porosity related to thermal degradation of the powder in relation to subsequent printing cycles.

## 2. Materials and Methods

Spherical gas-atomized powder of commercial Ti6Al4V was used as the base powder. The base powder was applied in two forms: (i) FRESH and (ii) USED for 12 sequential printing processes. The experimental set consisted of four kinds of samples: (i) singular lines; (ii) cubic samples printed using the same parameters used for the density check; (iii) tensile testing specimens and (iv) Charpy V-notched specimens (Charpy-V). Powder chemical composition was determined using different methods: Al content by inductively coupled plasma optical emission spectrometry; oxygen and nitrogen by hot extraction in helium using a LECO TCH 600 spectrometer (LECO Inc., St. Joseph, MI, USA); hydrogen by the inert gas fusion thermal conductivity method (JUWE H-Mat 2500 analyzer, JUWE Laborgeraete GmbH, Viersen, Germany); argon by mass spectrometry (IPI ESD 100) (InProcess Instruments GmbH, Bremen, Germany). A Horiba and Retsch CAMSIZER XT dynamic image analysis device (Retsch Technology GmbH, Haan, Germany) was used for measurement of particle size distribution. In order to investigate the melt pool characteristics, a series of single lines were printed with EOS EOSINT M280 LB-PBF equipment (EOS GmbH, Munich, Germany) using a laser (wavelength = 1064 nm) power of 240 W and a laser spot speed of 1650 mm/s, using a defocused laser beam for contour filling operation. The hatching distance was set to 500 μm. Single tracks were printed with an increasing powder bed depth: from 25 μm up to 115 μm. The aforementioned parameters were based on the pre-screening tests and selected to highlight the difference between FRESH and USED powders [28]. In total, 90 lines were printed using each powder condition. The single tracks were cut and their cross-sections were observed using a light optical microscope (LOM, Zeiss, Oberkochen, Germany) and then described with the following numerical parameters: area, height and width. Cubic, tensile and Charpy specimens were printed using the parameters listed in Table 1. The set of parameters was based on a previous study aimed at optimization of the printing parameters in respect to the density of reused Ti6Al4V powders [24]. The selected parameters were found to deliver 3D-printed components of nearly full relative density while highlighting the negative aspects of working with reused powders. The Charpy samples were printed in vertical position (*z*-axis). The printing strategy assumed that each sequential layer was rotated by 67° in relation to the previous one. All the samples were printed in *z*-axis (perpendicular to the XY printing plane). Directly after the manufacturing, the samples were subjected to a stress-relieving heat treatment as follows: heating up to 923 K at a rate of 20 K/min, then keeping an isothermal temperature for two hours followed by cooling down with the furnace. Argon of 99.999% (5.0 class) purity was used as a protective atmosphere. A density check was performed using a water displacement method and was repeated five times in accordance with ISO 3369:2006 standard [29].

Charpy tests using a 300-J hammer and notched 10 mm × 10 mm × 50 mm specimens were performed in accordance with ISO 148 [30]. Tensile tests were performed on cylindrical samples of a diameter of 6 mm using a gauge length of 30 mm and in accordance with ASTM E8 [31] at a traverse speed of 1 mm/min on a Zwick and Roell universal testing machine (ZwickRoell LP, Kennesaw, GA, USA). All tests were performed at room temperature.

The values of surface tension and density of the molten pieces of the printed Ti6Al4V tensile testing specimens were measured by the oscillating drop (OD) method in an electromagnetic levitation (EML) apparatus. A brief description of the measurement method is available in Appendix A, Appendix A.1. Technical details of the levitation setup at Graz University of Technology (TU Graz) were already discussed in the literature [32,33,34,35,36,37].

## 3. Results

### 3.1. Powder Characteristics, Melt Pool Behavior and Physicial Properties of the Melt

**Figure 1:**
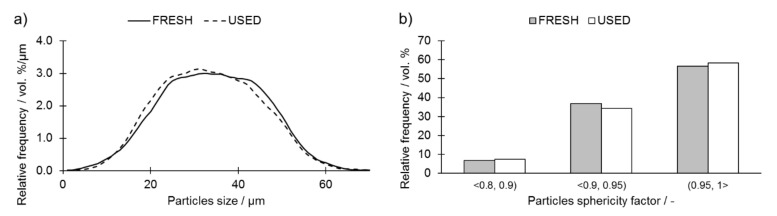
(**a**) particle size distribution and (**b**) particle sphericity histograms of the FRESH and the USED Ti6Al4V powders, highlighting the change in distribution of particle size and shape (in vol.%) in USED powder with respect to its FRESH counterpart.

The comparison of the particle size distribution of both powders presented in Figure 1 shows that the prolonged use of the Ti6Al4V resulted in a noticeable shift in the fraction distribution maximum in the direction of finer particles. This observation may suggest that the repetitive recycling of Ti6Al4V powder influences particle size distribution. This change occurs due to the interaction of the powder bed and the laser beam resulting in the creation of by-products, which, furthermore, can often interact with the powder bed, e.g., spatters, redeposits and condensate [17]. Powder particles with fused spatters or simply agglomerated by the laser heat are sieved out during the powder sieving step. As a result, a slightly finer powder is created after each recycling process. The analysis of the shape of the particles of both powders showed that, as presented in Figure 1b, a small shift in the sphericity factor towards higher values may be noticed in USED powder. This may indicate accumulation of spherical by-products of laser–powder interaction (e.g., spatters) [17,38].

**Figure 2:**
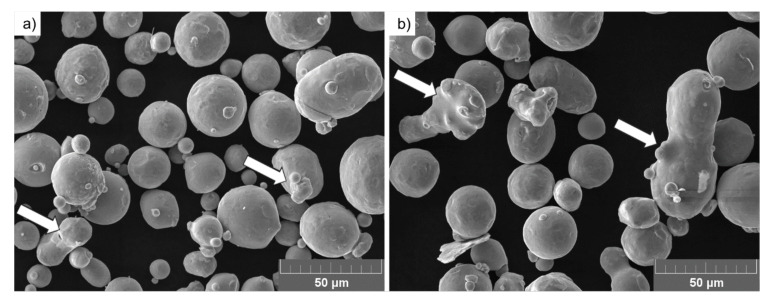
SEM micrographs of the Ti6Al4V powders: (**a**) FRESH powder showing particles with satellites (marked with arrows) and (**b**) USED powder showing the appearance of irregular particles (marked with arrows).

Scanning electron microscope (SEM) micrographs presenting particles revealed the presence of particles with satellites (indicated with arrows in Figure 2a) and elongated particles of irregular shapes in both FRESH and USED tested powders (Figure 2).

The gaseous elements intake (due to reuse) was investigated and is presented in Table 2. Oxygen, nitrogen and hydrogen were present in higher concentrations in USED powder. The oxygen intake remained in good agreement with the observations of Quintana et al. [39], who reported slow and steady increases in both nitrogen and oxygen contents in reused titanium powder.

According to Santecchia et al. [40], when the time of flight of a spatter particle is long enough, its surface can experience strong oxidation. It was observed mostly in the case of highly reactive powders (i.e., Ti6Al4V) due to their chemical composition characterized by the high presence of elements with a high oxidation potential. Such an effect was already observed in powders of Al-Si10-Mg by Simonelli et al. [15]. Similar observations were also reported by Stutton et al. [41], who detected patches of oxides on reused AISI 304 powder.

**Figure 3:**
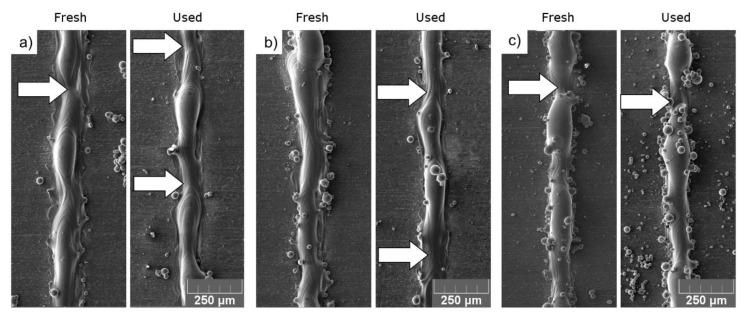
Representative SEM pictures of single tracks printed using FRESH and USED Ti6Al4V powder at a powder depth of (**a**) 25, (**b**) 60 and (**c**) 100 μm. Arrows indicate the spots where the deposited material is missing.

A comparison of representative single tracks produced with increasingly thick powder layers is presented in Figure 3. The single-track testing procedure was modified in the given case by applying a continuously increasing powder depth. Therefore, each sequential single track was printed on a powder layer deeper by one incremental step of 1.0 μm. This allowed the acquisition of information about the behavior of the melt track under close-to-real-life conditions, in which the powder bed depth varies due to the local waviness of the previous layer and local lack of powder.

**Figure 4:**
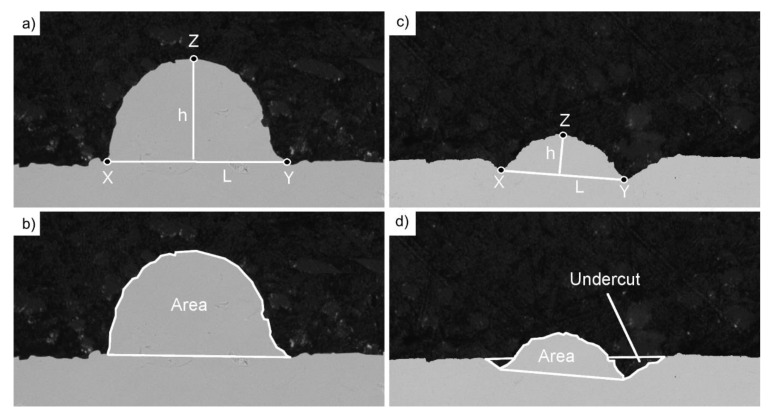
Measurement scheme of a single-track cross-section (STCS): (**a**) base length and height of a proper track; (**b**) area and perimeter of a proper track; (**c**) base length and height of a depleted track; (**d**) area and Undercut of a depleted track.

The visual observation did not bring a definitive answer to whether the reuse of a powder influences the quality of the melt track or not; therefore, cross-sections of all melt tracks were investigated. Subsequently, all the STCSs were measured in accordance with the scheme presented in Figure 4.

As indicated by arrows in Figure 3, the local accumulation of the material (mid-stage leading to balling phenomenon) was accompanied by unwanted transport of the molten material along the melt track axis. This further results in depleting certain regions of material, as presented in Figure 4c,d, referred to as Undercuts.

**Figure 5:**
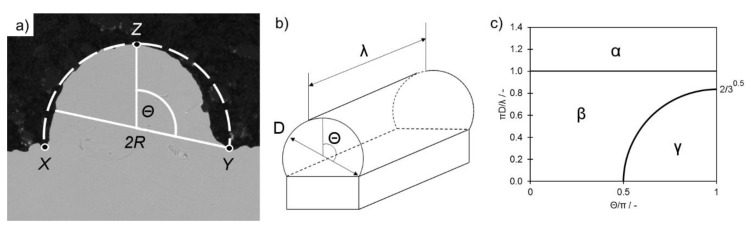
(**a**) Scheme of semi-cylinder measurement; (**b**) overview of the semi-cylinder approximation and (**c**) map of liquid cylinder stability. Based on Gusarov and Smurov [21].

Single-track experiments using LB-PBF [27] showed that the moving laser spot creates an elongated pool of molten metal in a quasi-semi-cylindrical cross-section due to the large influence of surface tension. The former plays a great role due to the size of a considered system, which typically does not exceed 100 μm (diameter of a melt pool). The stability of a melt pool is strongly dependent on the material properties, powder characteristics and process parameters [42]. The scheme of melt track is presented in Figure 5.

The symbols presented in Figure 5 represent the following: *λ*—length of the liquid semi-cylinder; *Θ*—angle between the vertical plane and the diameter perpendicular to the contact angle of the semi-cylinder; *D*—diameter; *α*— stable non-distorted semi-cylinder; *β*—distorted cylinder is stable while non-distorted becomes unstable; *γ*— unstable liquid semi-cylinder.

In accordance with the analysis performed in [21], the liquid semi-cylinder remains stable only when the series of conditions are fulfilled, which are mostly dependent on the melt pool length [21] (Equation (1)):

(1) $$\frac{\pi D}{\lambda }\;>\;1$$

When its shape becomes distorted, however, the cylinder shape changes to segmented and its stability condition is then expressed by Equation (2) [17]:

(2) $$\frac{\pi D}{\lambda }\;>\;\sqrt{\frac{2}{3}}$$

The boundary conditions expressed in Equations (1) and (2) take into consideration the length *λ* of a cylinder; however, for the segmented cylinder, there is also an additional one describing its stability independent of the cylinder length:

(3) $$\frac{\Theta }{\pi }\;>\;\frac{1}{2}$$

As the wetting angle between molten Ti6Al4V and its solid counterpart is a physical constant, the shape of the liquid cylinder cross-section should remain intact. As it emerges from the conditions (Equations (1)–(3)), the cylinder stability is mainly dependent on the *Θ* angle, which, under constant conditions, is directly dependent on the width of the surface supporting the cylinder.

When the liquid cylinder loses the stability condition, it becomes unstable and a “balling effect” phenomenon occurs. This results in the creation of “bulbs” of a solidified metal separated with regions depleted of metal, as observed in Figure 3c. Such structures are more stable due to lower overall surface energy [23].

The measurements of the STCS results are presented in Figure 5 in the “deposited area” function. This was intended to allow comparison between STCSs of similar dimensions, where:

(4) Deposited Area = Area − Undercut area

**Figure 6:**
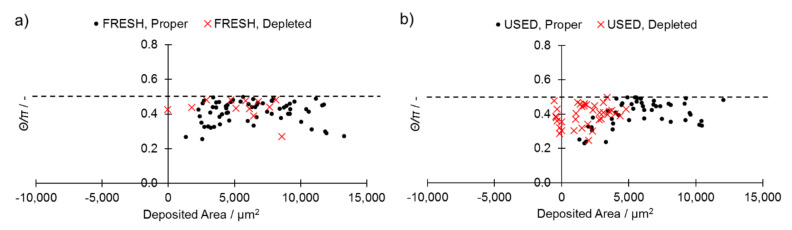
Comparison of proper and depleted STCSs’ shape parameters for (**a**) FRESH and (**b**) USED powders in function of area of STCS. The dashed line represents the stability condition presented in Equation (3).

Analysis of the stability parameter (Equation (3)) presented in Figure 6a,b shows that for the observed STCSs, its value always remains lower than 0.5. This means that all of the observed depleted STCSs remain stable regardless of the powder type (FRESH, USED), the powder depth and the melt pool length (Figure 5c).

The radius was calculated as the radius of an arc connecting the most distant points of length (L) and height (*x*, *y*, *z* presented in Figure 4a,c and Figure 5a).

Utilization of a USED powder led to an increase in the fraction of STCSs that were characterized by the presence of an Undercut (red crosses in Figure 6) up to 43.0% when compared with 16.0% for the FRESH powder. This suggests that prolonged utilization of a powder destabilizes the melt track through facilitating a balling effect and an axial flow of the molten metal along the single-track axis. The main difference between FRESH and USED powders was in the oxygen and nitrogen contents, whereby an increase was measured in USED powders (Table 2). Oxygen may effectively influence the stability of the melt pool by negatively altering the wetting conditions of the melt pool; this may subsequently result in higher values of the *Θ* angle, as well as in a collapse of the liquid cylinder by pushing it into the region lacking in stability γ (Figure 5). This shows how reuse of powder may have a detrimental influence on the melt track quality and lead to its increased instability during the 3D printing process. This finding may have an effect that is both crucial and detrimental on builds in which extraordinary precision is required, e.g., when lattice structures are produced [43].

As indicated in the literature [44,45], it is assumed that the presence of oxygen and interstitial hydrogen on the particles can impact the chemical composition of the melt pool and its surface tension, causing unfavorable wetting conditions and affecting the following solidification and densification of the metal part.

Measurements of density and surface tension of molten pieces of both FRESH and USED Ti6Al4V tensile test samples were performed to evaluate the observed changes in the melt pool behavior. The details of the sample and experiment preparation, experimental procedure, experiments, temperature calibration and uncertainty analysis can be obtained from Appendix A, Appendix A.2.

According to Keene [46], even slight increases (several ppm) in oxygen content in a molten metal may change the molten viscosity by as much as 10%. Based on Figure 5c and Equations (1)–(3), balling due to an axial flow of the molten metal along the melt track can occur when a semi-cylinder of the molten metal is too long in respect to the STCS dimensions. Furthermore, this means that if the *Θ* angle increases, then the maximum length of the stable melt track decreases. Since higher surface tension results in higher *Θ*, one may conclude that increasing the surface tension will result in promoting the balling effect.

Surface tension data were obtained from 1920 to 1995 K. Compared to the density measurements, slightly higher temperatures were reached since surface tension measurement allows the sample to have a lower levitation position (the side view is not needed) and, thus, experience a higher inductive heating power.

**Figure 7:**
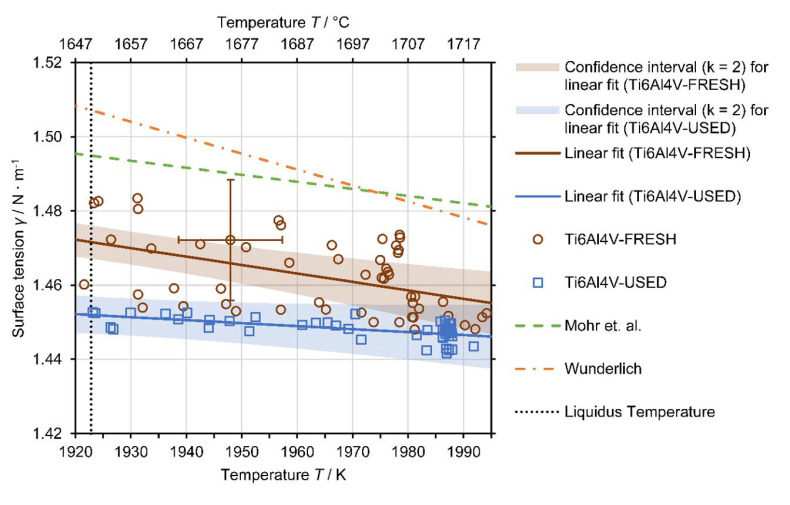
Results of the single surface tension measurements of FRESH (red circles) and USED (blue squares) Ti6Al4V, linear fits (solid red and blue lines) in accordance with the linear models (6), (7) and confidence intervals (k = 2, shaded red and blue areas) for the fits. Only one uncertainty bar indicating the typical measurement uncertainty of the individual data points is shown to facilitate better readability of the figure; see Appendix A.2.5 for more information about the uncertainty analysis. As a comparison, the reference data by Mohr et al. [49] (µ-g experiments on-board the International Space Station (ISS)) are plotted as a green dashed line and the average of two datasets by Wunderlich [50] (µ-g experiments on parabola flight) as an orange dash dotted line. The melting temperature of 1928 K by Boivineau et al. [47] is plotted as a dotted black line.

Figure 7 shows the obtained surface tension data of four experiments for USED Ti6Al4V and five experiments for FRESH Ti6Al4V. A linear model (5) describing the surface tension as a function of temperature $\gamma \left(T\right)$ was fitted to the surface tension data,

(5) $$\gamma \left(T\right)=\gamma_{L}+\frac{\partial \gamma }{\partial T}\left(T-T_{L}\right)$$

where $\gamma_{L}$ denotes the surface tension at the liquidus temperature, $\frac{\partial \gamma }{\partial T}$ is the change in surface tension with temperature *T* and *T*_L_ is the liquidus temperature of 1928 K (adopted from Boivineau et al. [47]).

The surface tension data indicate a small difference in surface tension between USED and FRESH samples. The single experiments of USED show good agreement with each other, but the surface tension data of the single experiments of FRESH not only show a larger scatter, but they are also at slightly higher surface tension values when compared to the USED data. Although the observed difference in surface tension between USED and FRESH is in the order of measurement uncertainty, which is partly overlapping, result clustering of a kind can be found for both materials at two marginally different surface tension ranges. Therefore, a separate fit for USED and FRESH was implemented ($\gamma_{\mathrm{USED}}\left(T\right)$ and $\gamma_{\mathrm{FRESH}}\left(T\right)$, respectively), and the resulting fit equations are:

(6) $$\gamma_{\mathrm{USED}}\left(T\right)\;=\;\left(1.452\;\pm \;0.005\right)\;\frac{N}{m}\;+\;\left(-0.08\pm 0.11\right)\;\times \;10^{-3}\frac{N}{m\cdot K}\;\times \;\left(T-1928\;K\right)$$

(7) $$\gamma_{\mathrm{FRESH}}\left(T\right)\;=\;\left(1.470\;\pm \;0.004\right)\;\frac{N}{m}\;+\;\left(-0.23\pm 0.11\right)\;\times \;10^{-3}\frac{N}{m\cdot K}\;\times \;\left(T-1928\;K\right)$$

In accordance with the model coefficients of Equations (6) and (7), there is only a small difference in surface tension at the liquidus temperature between FRESH and USED of 0.018 N∙m^−1^ (1.2%). However, the surface tension temperature dependence (gradient) of USED is less steep downwards by 0.15 × 10^−3^ N∙m^−1^∙K^−1^ (65%) compared to FRESH, yet the numeric value of the deviation should be interpreted with caution due to the large uncertainty of this parameter for both fits. The observed difference in the temperature dependence of the surface tension is particularly interesting because Zhao et al. [48] showed that instabilities of the melt pool can be induced by the Marangoni convection, which is caused by the temperature dependence of surface tension.

To facilitate the assessment of the data, Figure 7 shows additional reference data for the surface tension of Ti6Al4V from the literature: Mohr et al. [49] recently published data obtained with the electromagnetic levitation facility onboard the International Space Station (ISS). Due to the microgravity (µ-g) conditions onboard the ISS, only weak positioning forces and, thus, lower field strengths are needed. As a result, the data quality is improved compared to terrestrial levitation experiments. The data of Wunderlich [50] were obtained in levitation experiments onboard a parabolic flight where reduced µ-g conditions were established for a short duration during a parabola. Wunderlich’s data originate from experiments with samples of two different oxygen concentrations; however, the data showed good agreement for both oxygen concentrations within measurement uncertainty.

The surface tension measurement results obtained in this study are in good agreement with the reference data from the literature. The slight deviation to lower surface tension values is attributed to the fundamental difference in the raw sample materials. Although detailed information about the supplier or the purity of the sample material is missing in the referenced publications, it can be assumed that the raw sample material was manufactured using a traditional production route (e.g., casting), whereas the sample material in this study originated from selective laser melting of a powder which has a large surface area. The powder itself was produced by gas atomization. Therefore, parts printed from the powder are inherently prone to exhibit higher levels of oxygen concentration compared to conventional manufactured parts, in which the surface tension is potentially lowered.

Slight deviations may, in principle, also arise from the differences in the experimental setup and especially the boundary conditions, e.g., differences in the size of the samples and their deformation amplitude. A recent study by Xiao et al. [51] showed that as the small oscillation condition of the Rayleigh equation used in the oscillating drop method is violated, nonlinear effects lead to lowered surface tension measurement results. However, this effect is considered negligible as the observed oscillations in this study were very small; Mohr et al. state the same for their data.

Density data were obtained from the undercooled regime of 1855 K up to the slightly superheated regime of 1975 K. It was not possible to acquire density data at higher temperatures since the inductive heating power was limited. The only way to increase the inductive heating power was to lower the samples’ levitation position, but then the side-view projection would already be partly covered by the levitation coil and, thus, volume measurement would no longer be possible.

**Figure 8:**
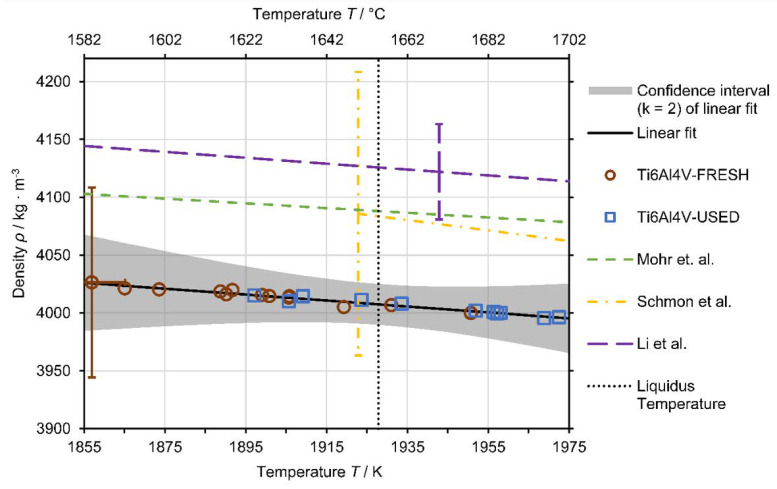
Results of the single density measurements of the FRESH Ti6Al4V in red circles and USED Ti6Al4V in blue squares. The measurement uncertainty for the individual data points is represented by only one uncertainty bar to facilitate better readability of the figure. The solid black line shows the linear fit of all data in accordance with the model (9), the confidence interval (k = 2) for the fit is the shaded grey area (see Appendix A.2.5 for more information about the uncertainty analysis). As a comparison, reference data by Mohr et al. [49] (µ-g experiment on-board the ISS) are plotted as a green dashed line, (extrapolated) data by Schmon et al. [52] (exploding wire technique) as a yellow dash dotted line and data by Li et al. [53] (electrostatic levitation) as a long-dash purple line. Their respective uncertainty estimates (if given) are visualized by the corresponding uncertainty bars. The liquidus temperature of 1928 K by Boivineau et al. [47] is plotted as a black dotted line.

Figure 8 shows the obtained density data of four experiments for USED and three experiments for FRESH Ti6Al4V. A linear model (Equation (8)) describing the density as a function of temperature $\rho \left(T\right)$ was fitted to the data:

(8) $$\rho \left(T\right)=\rho_{L}+\frac{\partial \rho }{\partial T}\left(T-T_{L}\right)$$

where $\rho_{L}$ denotes the density at the liquidus temperature, $\frac{\partial \rho }{\partial T}$ is the change in density with temperature *T* and *T*_L_ is the liquidus temperature. Since no significant difference in density data between USED and FRESH was observed and all single experiments showed a good agreement with each other, the model was fitted to all density data. The resulting model equation is:

(9) $$\rho \left(T\right)=\left(4007\pm 18\right)\;\frac{{\mathrm{kg}}^{\;}}{m^{3}}+\left(-0.26\pm 0.52\;\right)\frac{{\mathrm{kg}}^{\;}}{m^{3}\cdot K}\cdot \left(T-1928\;K\right)$$

Figure 8 also includes reference data for liquid density from the literature. The recent data by Mohr et al. [49] were obtained in µ-g experiments onboard the ISS. Schmon et al. [52] measured the liquid density of Ti6Al4V with the exploding wire technique. They report an expanded uncertainty for their data of ±3%, which is also depicted in Figure 8. Another reference shown is the data by Li et al. [53] that were obtained using an electrostatic levitation apparatus and which report an uncertainty of ±1%.

The measured density data show good agreement with the reference data within measurement uncertainty. The difference of the density at the liquidus temperature determined in this work compared to the values reported in the literature follows: Mohr et al.: −80 kg∙m^−3^ (−2.0%); Schmon et al.: −79 kg∙m^−3^ (−1.9%); Li et al.: −115 kg∙m^−3^ (−2.8%).

### 3.2. Material Strucutre and Mechanical Properties

**Figure 9:**
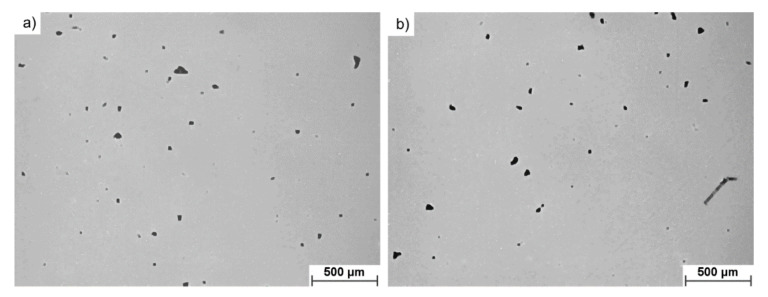
Representative micrographs of porosity of investigated materials, (**a**) FRESH and (**b**) USED Ti6Al4V, showing no apparent difference in porosity morphology between investigated samples.

The analysis of porosity performed using LOM showed that the pores in both investigated materials were characterized by similar distributions with no apparent preference of occurrence or clustering. The observed pores were not interconnected and their shapes were rounded (Figure 9).

**Figure 10:**
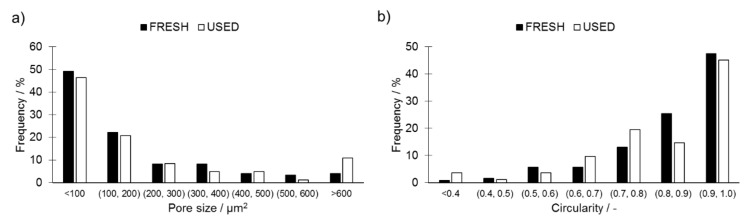
Comparison of (**a**) pore size distribution and (**b**) aspect ratio distribution in cubes printed from FRESH and USED Ti6Al4V powders, highlighting the appearance of less circular pores in samples 3D printed using USED Ti6Al4V powder.

The qualitative analysis of porosity performed using image analysis showed that prolonged use of the investigated powder results in a noticeable shift in their size distribution (Figure 10a). Pores observed in samples manufactured using FRESH powder were of a smaller size compared to those observed in samples manufactured from USED powder. Furthermore, in USED samples, occurrence of larger pores (>600 μm^2^) was more pronounced since their share increased to 11.0% from the 4.1% observed for samples produced from FRESH powder. This is attributed to the instability of a melt track when USED powder is utilized. This lack of stability creates an uneven printed surface and the powder layer laid on top of this will, thus, naturally also vary in depth. Since the laser depth penetration power was limited, the localized patches of powder layer were also limited. This interaction resulted in the occurrence of less round pores in samples produced from USED powder (which showed higher instability of melt track).

The analysis of the pore shapes presented in Figure 10b shows that USED powder utilization resulted in the occurrence of pores characterized by more developed perimeters. The average pore morphology observed in samples produced from FRESH powder was characterized with values of size and circularity of (90 ± 17) μm^2^ and 1.53 ± 0.06, respectively, while for the USED counterparts, these values were equal to (119 ± 24) μm^2^ and 1.66 ± 0.09. The circularity parameter was applied, which is expressed by Equation (10):

(10) $$\mathrm{circularity}\;=\;4\pi \left(\mathrm{area}/{\mathrm{perimeter}}^{2}\right)$$

The overall porosity present in the manufactured samples—evaluated using a water displacement method in accordance with ISO 3369:2006 [29]—showed that the samples manufactured using USED powder displayed a slightly higher relative density (99.18% ± 0.16%) than that for the FRESH powder (99.08% ± 0.14%). These results suggest that there is no distinguishable difference between the two tested materials in terms of porosity.

**Figure 11:**
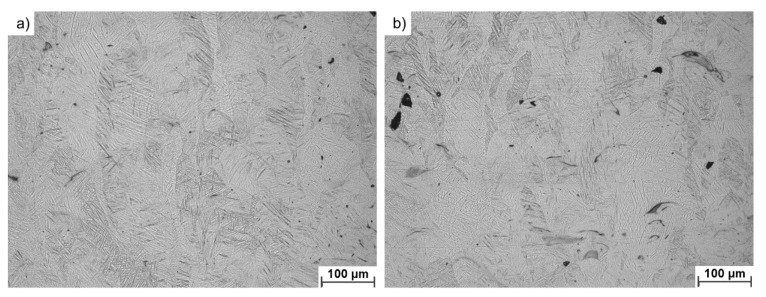
Representative micrographs of investigated 3D-printed materials: (**a**) FRESH and (**b**) USED powder showing no visual microstructural differences.

Figure 11 presents a more detailed microstructural analysis showing the presence of a typical microstructure of Ti6Al4V alloy manufactured via LB-PBF and subsequently stress-relieved: long columnar grains of martensite originating from the epitaxial growth, a common microstructural feature in this alloy. There are no apparent visual differences in the phase composition between samples manufactured using USED and FRESH powders. The performed annealing process was aimed at relieving stresses rather than to decompose martensite. According to Ghods et al. [38], the chemical changes observed in the USED powder would require a greater increase in the oxygen content in order to have a noticeable impact on the microstructure of a 3D-printed Ti6Al4V.

**Figure 12:**
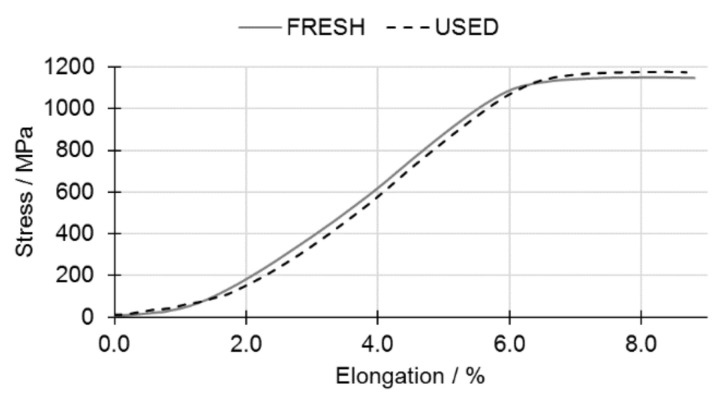
Comparison of the tensile behavior of 3D-printed samples from FRESH Ti6Al4V powder (solid line) and USED Ti6Al4V powder (dashed line).

**Figure 13:**
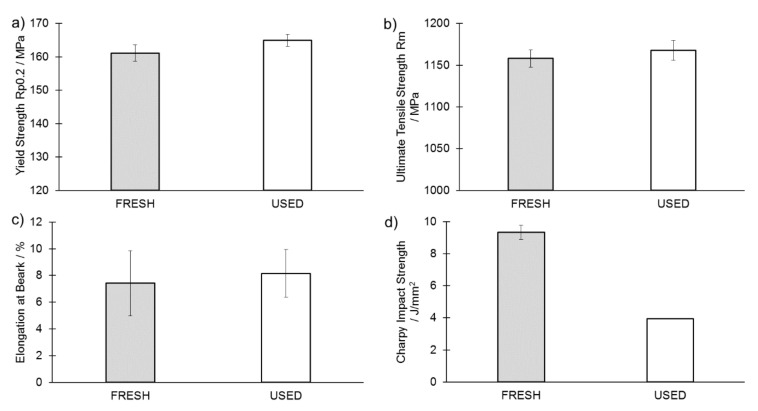
Comparison of properties of samples produced from FRESH and USED Ti6Al4V powders: (**a**) Yield strength; (**b**) tensile strength, (**c**) elongation at break and (**d**) Charpy-V impact strength.

The results of mechanical testing are presented in Figure 12 and Figure 13. It can be observed that values of both offset yield strength (R_p0.2_) and ultimate tensile strength (Rm) are slightly higher in samples produced from USED powder. This is in good agreement with the findings of the other researchers, as presented in Table 3 [5,39,54,55]. Such an increase may be attributed to the higher oxygen content, which was found to cause lattice straining and to hinder the dislocations slippage. Increased presence of oxygen, however, does not reduce the elongation at break (ε) as measured in tensile test static—an observable change would require a much larger oxygen content [56,57]. A higher absorbed nitrogen content is also believed to partially contribute to the observed change of R_p0.2_ and Rm presented in Figure 12 and Figure 13. The analysis of averaged values accompanied by standard deviation shows that slight changes of R_p0.2,_ in opposition to Rm and ε seem to be relevant.

**Figure 14:**
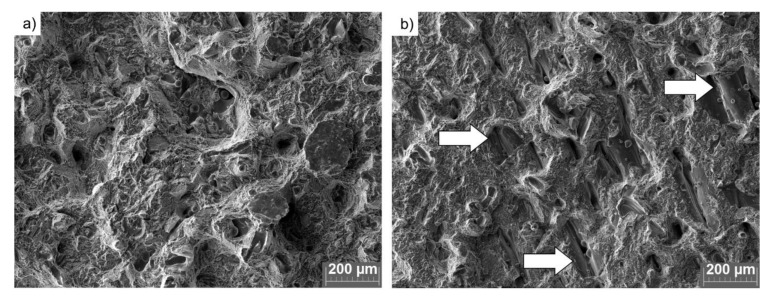
SEM photographs of fracture surfaces of tensile samples: (**a**) FRESH and (**b**) USED powders. The arrows indicate lack of fusion.

**Figure 15:**
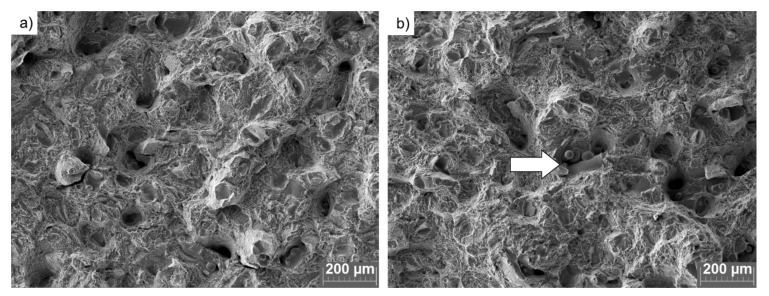
SEM photographs of fracture surfaces of Charpy-V samples: (**a**) FRESH and (**b**) USED powders. The arrow indicates the lack of fusion.

The major difference between the two analyzed sample sets was noticed in the impact strength values, which decreased by over 50% from (9.4 ± 0.5) J/cm^2^ for FRESH samples to (4.0 ± 0.1) J/cm^2^ for the samples produced from USED powder (Figure 13d). Based on the observations made in combination with the fracture surfaces presented in Figure 14 and Figure 15, the observed decrease in resilience should most likely be attributed to the lack of fusion [58]. Lack of fusion between sequential layers was already observed by Zhao et al. [16] and the appearance of this was also attributed to the appearance of fewer spherical particles in the reused feedstock powder. The observed extensive lack of fusion (Figure 14b) is believed to originate from the axial flow of the melt along the melt track axis due to unfavorable wetting conditions created by the presence of oxidized particles.

## 4. Conclusions

Two types of the same Ti6Al4V powder were compared, with a focus on their ability to create a stable molten pool in the LB-PBF process. It was found that the repetitive use of Ti6Al4V increases the content of gaseous elements (oxygen and nitrogen) in the powder and causes the appearance of finer and more spherical particles with respect to the FRESH powder. Both of the observed effects take place due to the continuously increasing content of by-products (e.g., spatters) caused by repeated processing and interaction with a laser beam. Larger by-products (e.g., re-deposited particles and fused particles) are removed during the sieving step.

Furthermore, it was found that the recycled powder limits the wetting conditions of the melt track, resulting in its destabilization followed by an axial flow of the melt along the melt track axis. This leads to the appearance of localized areas depleted of deposited material. During the placement of the sequential layer, this leads to the occurrence of uneven thickness in the sequential powder layer that is too thick for the laser to fully melt, which, in turn, leads to a lack of fusion. Consequently, the lack of fusion results in the appearance of larger and less round porosity. The porosity analysis showed that the reuse of Ti6Al4V powder leads to a change of quality in the observed porosity—pores observed in reused powder were, on average, larger (119 μm^2^ ± 24 μm^2^) than those observed in samples produced from the fresh material (90 μm^2^ ± 17 μm^2^). They were also of more irregular shapes—on average, the circularity parameter changed from 1.53 ± 0.06 for samples produced from fresh material to 1.66 ± 0.09 for the counterparts produced from reused material. This change was attributed to the observed change in powder shape: particles of the USED powder were of more irregular shapes. Combination of more irregular porosity and patches where fusion between layers is missing creates a path of easy crack propagation. Because of this, a significant decrease (by over 50%) in the Charpy impact strength was observed in samples produced from the reused powder.

Such detrimental changes were not observed in other mechanical properties, i.e., R_p0.2_, Rm and ε. Multiple reuse of a Ti6Al4V powder leads to slight increase in the R_p0.2_ with respect to the fresh powder due to the increased oxygen content which anchors the dislocations and increases the lattice strain. The values of ε and Rm remained virtually unchanged.

Experiments performed using electromagnetic levitation showed that the changes in surface tension are in a range approaching the detectable range for the observed change in the content of gaseous elements. While the difference between samples from the reused and the fresh powder regarding the surface tension at the liquidus temperature was very small (−1.2%), the difference in the temperature dependence of surface tension was more pronounced. As the surface tension temperature dependence is the driving force for the Marangoni effect, this can result in different melt pool stabilities influenced by this effect [48].

Use of the Charpy impact strength test is recommended to monitor the quality of reused Ti6Al4V powder since the other techniques were shown to be lacking adequate sensitivity to highlight all the negative changes occurring in the reused feedstock.

## Figures and Tables

**Table 1:** Parameters used for printing bulk samples.

Hatching Distance/μm	Laser Power/W	Laser Speed/mm·s^−1^	E Linear/J·mm^−1^	E Area/J·mm^−2^	E Volume/J·mm^−3^
120	240	1650	0.145	1.212	40.4

**Table 2:** Content of elements in investigated powders.

Content	Al/wt %	O/wt %	N/wt %	Ar/ppm	H/ppm
FRESH	6.13	0.16	0.0281	1.1	21
USED	6.40	0.18	0.0358	1.0	23

Error: Al ± 0.33; O ± 0.006, N ± 0.0044.

**Table 3:** Comparison of mechanical properties with the state of the art. Sample printing orientation: vertical in respect to the horizontal powder plane.

Source	Reuse Times (Max)	Charpy-V	R_p0.2_	Rm	ε	Heat Treatment
		J/cm^2^	MPa	MPa	%	
This study, max value	0	9.4 ± 0.5	1111 ± 14	1158 ± 8	7.1 ± 1.2	923 K, 2 h
This study, max value	12	4.0 ± 0.1	1137 ± 9	1168 ± 9	8.2 ± 1.6	923 K, 2 h
[52]	0	N/A	1132 ± 13	1156 ± 13	8 ± 0.4	923 K, 4 h
[5]	0	N/A	1112 ± 3	1165 ± 2	11.6 ± 1.2	As built, modified inter-layer time.
[39]	0	N/A	878.7 ± 7.6	984.3 ± 0.6	13.7 ± 0.6	Subjected to isostatic pressing
[39]	31	N/A	881.0 ± 3.6	1002.7 ± 1.2	14.7 ± 0.6	Subjected to isostatic pressing
[55]	0	N/A	N/A	1030	N/A	-
[55]	12	N/A	N/A	1101	N/A	-

**Figure A1:**
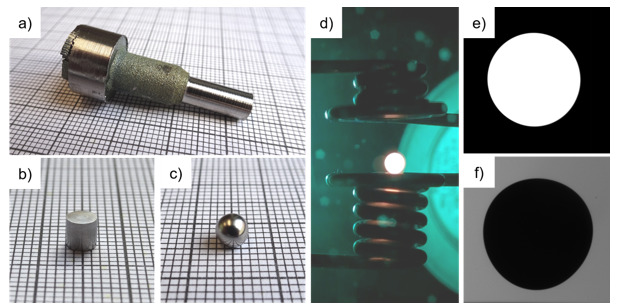
Pictures of the electromagnetic levitation (EML) samples (on graph paper) and the experiment itself. (**a**) Tensile test sample machined with a lathe, (**b**) prepared sample (small cylinder) cut from the tensile test sample, (**c**) sample after levitation, (**d**) sample during levitation (with background illumination), (**e**) image from surface tension measurement (top view) and (**f**) image from density measurement (side view).

**Figure A2:**
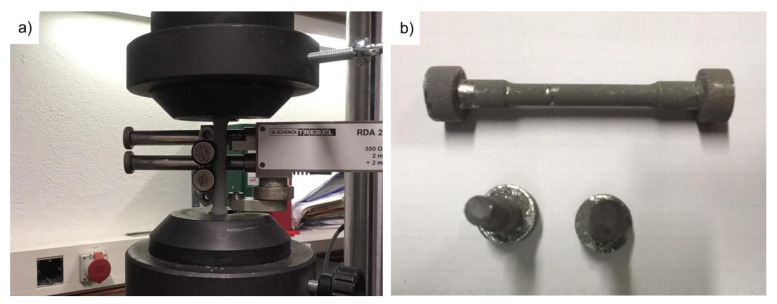
Pictures of 3D-printed tensile strength samples from Ti6Al4V powder: (**a**) overview of the test setup; (**b**) the sample overview after the test.

## Data Availability

The data presented in this study are available on request from the corresponding author.

## Appendix A

### Appendix A.1. Electromagnetic Levitation (EML)

In an EML device, the sample is freely levitated in space, only environed by an inert gas atmosphere, and thus, contact- and containerless conditions are ensured throughout the entire experiment. This is an advantage over other conventional surface tension measurement methods that are mostly contact-based and thus suffer from problems that arise with the high chemical reactivity of molten metals and alloys.

Levitation is achieved by applying an inhomogeneous, radiofrequency (350 kHz) electromagnetic field to the sample that exerts a Lorentz force on it, counteracting gravitational force. The electromagnetic field is generated by a levitation coil (inductance *L*) that forms, together with a capacitance (*C*), an *LC* oscillating circuit which is fed by a high-frequency generator. At the same time, the sample is heated inductively by the ohmic losses of the induced eddy currents in the sample material up to the melting temperature and beyond that.

When the sample material is molten, oscillations of the sample surface can be observed. Using the OD method based on Equation (A1) by Lord Rayleigh [59], surface tension *γ* can be deduced from the surface oscillations’ angular frequency *ω*_R_ (also known as the Rayleigh frequency) of the liquid droplet of mass *M*:

(A1) $$\omega_{R}^{2}=\frac{32\pi }{3}\cdot \frac{\gamma }{M}$$

The surface oscillations and the shape of the sample are recorded by high-speed cameras and analyzed further by an edge-detection algorithm to deduce a frequency spectrum of the samples’ surface oscillations. This frequency spectrum is then used to identify the frequencies of the oscillation modes. Finally, the Cummings and Blackburn [60] correction for terrestrial conditions must be applied to the Rayleigh Equation (A1) to determine the surface tension from the observed samples’ oscillation modes.

Liquid density is determined by dividing the weighed mass through the measured samples’ volume. Assuming vertical axis symmetry, the recorded two-dimensional side-view projection is used to deduce the three-dimensional volume of the levitated sample.

Temperature measurement is performed with a contactless method using a single-wavelength pyrometer (IMPAC IGA6 Advanced, LumaSense, Santa Clara, CA, USA). Since the normal spectral emissivity of the sample material at the pyrometer wavelength is usually unknown beforehand, the pyrometer is initially calibrated at a reference temperature (e.g., melting temperature) and the measured temperature is corrected, assuming the emissivity to be constant in the liquid phase.

For further details about EML, the OD method and the subsequent data evaluation in general and the levitation setup at TU Graz in particular, the interested reader is referred to the publications [61] and [32,33,34,35,36,37] since this information would go beyond the scope of this paper.

### Appendix A.2. EML Experimental Details

#### Appendix A.2.1. Sample and Experiment Preparation

For the EML experiments, small cylinders in the mass range of 110 mg to 163 mg were prepared and cut off from the FRESH and USED tensile test samples using a lathe. The surface of the cylinders was further cleaned with abrasive paper, followed by an ultrasonic bath in isopropyl alcohol for 10 min.

The samples were weighed on a balance (Mettler Toledo AB104-S-A, Greifensee, Switzerland) before being inserted into the EML processing chamber, which was then evacuated to a pressure of the order of 10^−6^ mbar to remove as much oxygen as possible from the processing chamber.

#### Appendix A.2.2. Experiment Procedure

During the experiment, the processing chamber was backfilled with a high-purity (N6.0, 99.9999 vol.%) gas mixture of argon with 4 vol.% hydrogen addition (Air Liquide custom gas mixture, Graz-Messendorf, Austria) up to a pressure of 700 mbar. Temperature regulation of the sample was achieved by directing a stream of the gas mixture from below directly on the sample. To be able to solidify the sample at the end of the experiment, the cooling gas stream was switched to a high-purity (N5.0, 99.999 vol.%) gas mixture of helium with a 4 vol.% hydrogen addition (Air Liquide custom gas mixture). The purpose of the hydrogen addition in both gas mixtures was to prevent possible oxide formation due to residual oxygen in the process gases and the processing chamber, respectively.

At the beginning of each experiment, when the levitation was started, the sample was heated from room temperature up to the melting point. After melting, the sample was slightly superheated to ensure that the droplet is fully molten and that the alloy is homogeneous.

Surface tension and density measurements were then executed by recording high-speed videos while keeping the temperature constant during the video acquisition. By varying the cooling gas stream, the temperature of the sample was adjusted and the measurement procedure was repeated for different temperatures to measure surface tension and density as a function of temperature.

At the end of the experiment, the sample was first solidified by a strong cooling gas stream and then immediately landed on the sample holder. After opening the processing chamber, the sample was weighed again on the balance to determine possible mass loss during levitation.

#### Appendix A.2.3. Experiments

All levitation experiments that were successful are listed in Table A1, including the mass of each sample before and after the levitation experiment and the observed mass loss. The mass loss was, in general, very low—for some of the samples, as low as in the range of the repeatability (0.1 mg) of the balance used. The maximum mass loss was found for the experiment “Ti6Al4V-fresh_10” with a relative mass loss of only 0.56%. Therefore, all experiments listed in Table A1 were included in the data evaluation.

**Table A1:** List of levitation experiments that were successful and the corresponding mass before and after the experiment.

USED				FRESH			
exp.	*m*_start_/mg	*m*_end_/mg	Δ*m*/mg	exp.	*m*_start_/mg	*m*_end_/mg	Δ*m*/mg
Ti6Al4V-used_1	128.5	128.5	0.0	Ti6Al4V-fresh_1	135.8	135.8	0.0
Ti6Al4V-used_2	119.9	119.8	−0.1	Ti6Al4V-fresh_5	126.4	126.0	−0.4
Ti6Al4V-used_3	130.8	130.6	−0.2	Ti6Al4V-fresh_6	152.2	151.9	−0.3
Ti6Al4V-used_4	110.5	110.5	0.0	Ti6Al4V-fresh_7	163.3	163.0	−0.3
Ti6Al4V-used_5	148.5	147.9	−0.6	Ti6Al4V-fresh_8	151.8	151.6	−0.2
Ti6Al4V-used_6	154.2	153.9	−0.3	Ti6Al4V-fresh_9	146.4	146.3	−0.1
Ti6Al4V-used_7	160.9	160.7	−0.2	Ti6Al4V-fresh_10	160.4	159.5	−0.9
Ti6Al4V-used_8	157.9	157.8	−0.1	Ti6Al4V-fresh_11	132.2	132.1	−0.1

exp.: experiment number; *m_start_:* mass before levitation experiment (± 0.1 mg); *m*_end_: mass after levitation experiment (± 0.1 mg); Δ*m*: mass difference (± 0.16 mg).

#### Appendix A.2.4. Temperature Calibration

As elaborated in Appendix A (Appendix A.1), the temperature cannot be measured absolutely, and thus, the pyrometer must be calibrated at a reference temperature for each experiment. For this purpose, the liquidus temperature was chosen since the melting transition was clearly visible in the pyrometer time series data.

Since no data for the liquidus temperatures of USED and FRESH were available, a reference value for the liquidus temperature of Ti6Al4V of 1928 K published by Boivineau et al. [47] was assigned to both materials. This liquidus temperature was measured by the Thermo- and Metalphysics group of the Institute of Experimental Physics (IEP), Graz University of Technology (TU Graz), using the ohmic pulse-heating setup in combination with a “division of amplitude polarimeter”, and uncertainty was specified with ± 4%.

The absolute temperature values in the results presented are, thus, in need of careful interpretation, since the data will shift to lower or higher temperatures if the true liquidus temperature of USED and FRESH Ti6Al4V is lower or higher. However, for the main goal of this investigation, the comparison between USED and FRESH Ti6Al4V, this is irrelevant provided that there is no significant difference in the melting temperature of the two materials.

#### Appendix A.2.5. Uncertainty Analysis

The uncertainty analysis of the data presented was performed in accordance with the “Guide to the expression of uncertainty in measurement” (GUM) [62]. The stated uncertainties are expanded uncertainties at a 95% confidence level (coverage factor k = 2). The coefficients of the model Equations (6), (7) and (9) were determined following the guide by Matus [63] to account for the abscissa and ordinate uncertainties of the individual data points. For details on the uncertainty budget of the levitation setup at TU Graz, we refer to the publications [32,52,64] which include an in-depth description of this topic.

The uncertainty of the “true” liquidus temperature as described in Appendix A.2.4 was not included in the temperature uncertainty of the individual data points. An error in the reference liquidus temperature would simply lead to a shift of all measurement data to lower or higher temperature values (as elaborated in Appendix A.2.4), and therefore, an accordingly larger uncertainty in the temperature of the individual data points would not only be an inappropriate representation of this issue but would also give the (visual) impression of data quality being poorer than it actually is.
